# Does Streptococcus Salivarius Strain M18 Assumption Make Black Stains Disappear in Children?

**DOI:** 10.3290/j.ohpd.a43359

**Published:** 2020-02-12

**Authors:** Elena Bardellini, Francesca Amadori, Emanuela Gobbi, Anna Ferri, Giulio Conti, Alessandra Majorana

**Affiliations:** a Associate Professor, Department of Medical and Surgical Specialities, Radiological Sciences and Public Health, Dental School, University of Brescia, Brescia, Italy. Wrote the manuscript.; b Fellowship, Department of Medical and Surgical Specialities, Radiological Sciences and Public Health, Dental School, University of Brescia, Brescia, Italy. Collected the data and drafted the manuscript.; c Associate Professor, Department of Molecular and Translational Medicine, PiMiAA, University of Brescia, Brescia, Italy. Revised the manuscript for microbiological content.; d Fellowship, Department of Medical and Surgical Specialities, Radiological Sciences and Public Health, Dental School, University of Brescia, Brescia, Italy. Collected the data and helped to draft the manuscript.; e Fellowship, Department of Oral Surgery, University Vita-Salute San Raffaele, Milan, Italy. Collected the data and helped with the data analysis.; f Full Professor, Department of Medical and Surgical Specialities, Radiological Sciences and Public Health, Dental School, University of Brescia, Brescia, Italy. Revised the manuscript for intellectual content.

**Keywords:** child, pigmentation, probiotics, teeth

## Abstract

**Purpose:**

This randomised controlled study evaluated the effectiveness of an oral probiotic, *Streptococcus salivarius* M18 (SsM18), in children with black stains (BSs) in order to counteract their reformation.

**Materials and Methods:**

Fifty-eight children (aged 4–10 years) presenting with BSs were enrolled. They were randomly divided into two groups: group A (n = 29) included children who were given the test product containing SsM18 once a day for 3 months; group B (n = 29) included children who did not receive any treatment. Before beginning the study, all the children underwent professional removal of BSs. The assessment of BSs was done after 3 months (T1) and after 6 months (T2).

**Results:**

Four patients (1 belonging to group A and 3 to group B) were excluded from the study because they started antibiotic therapy. After 3 months (T1), BSs were detected in 6 of the 28 children (21.2%) from group A and in 13 out of the 26 (50%) children from group B (p < 0.05). After 6 months (T2), BSs were detected in 9 out of the 28 (32.1%) children from group A and in 14 of the 26 (53.8%) children from group B (p > 0.05).

**Conclusions:**

BSs formation in children could be prevented by administering *S. salivarius* M18.

Black extrinsic discolouration is a common clinical and aesthetic problem in childhood.^10,11,14–16,18^ Both primary and permanent teeth can be affected, with a reported prevalence of 1–20%.^17,21^ Black stains (BSs) are hard to remove by simply brushing one’s teeth, and tend to reform after professional cleaning. The mechanism of BS formation is still unclear. BSs contain insoluble ferric salt, most likely a ferric sulphide, and has a high content of calcium and phosphate.^19,20^ Various studies have reported higher consumptions of foods rich in iron, such as vegetables, dairy products and eggs, or water with a high concentration of iron in children with BSs. Early ultrastructural examinations of BSs demonstrated that these deposits consist of microorganisms embedded in an intermicrobial matrix.^25^ Chromogenic Gram-positive bacteria are currently considered the most important aetiological factor in the production of black pigmentation. The ferric sulphide might be formed by the reaction between hydrogen sulphide, produced by bacterial activity and the iron present in saliva or gingival exudates. Traditional bacteriological examinations have implicated *Actinomycetes* as the predominant cultivable microorganism found in BSs. Li et al^17^ performed a study using next-generation sequencing of the bacterial *16S rRNA* gene to evaluate the oral microbiota in children with and without BSs. Children with BSs had a significantly lower salivary microbiota diversity compared to other children. *Actinomyces* were more abundant in plaque samples of children with BSs. Alterations in quantity of other species might provide an environment that enables BS formation and deposition onto the enamel surface. The application of oral probiotics to help restore a balanced microbiota and thereby improve oral health is a relatively new concept. *Streptococcus salivarius* M18 (SsM18) (IDA classification: DSM 14865),^7,8^ a strain originally isolated from a healthy female subject during a specific search for an oral commensal strain capable of inhibiting mutans streptococci, has subsequently been shown to have a relatively broad spectrum bacteriocin-like inhibitory substance (BLIS) activity against *S. mutans* and *S. sobrinus*. It also produces both dextranase and urease enzymes which can respectively reduce plaque accumulation and its acidification. Recent clinical trials have revealed, along with its safety and tolerability profiles, SsM18’s ability to colonise and remain in the human oral cavity,^6,8^ where it lowers *S. mutans* counts in primary-school-aged children and reduces severe gingivitis and periodontitis in adults.^5,8^

## Materials and Methods

### Sample Selection

Children between the ages of 4 and 10 years who presented with extrinsic black teeth stains on examination at the Pediatric Dentistry Department of the Dental Clinic of the University of Brescia, in the period from January 2016 to December 2016, were enrolled. BSs were evaluated based on the presence of pigmented dark lines parallel to the gingival margin or an incomplete coalescence of dark dots, rarely extending beyond the cervical third of the crown.^4,13,18^ The exclusion criteria were oral diseases (eg, dental caries, periodontitis, salivary gland disorders, oral mucosal diseases, and others), orthodontic treatment, chronic diseases (eg, respiratory diseases, diabetes, epilepsy, celiac diseases), pharmacologic treatment (eg, antibiotics, corticosteroids). Occasional use of paracetamol or ibuprofen for fever or pain control was allowed.

### Study Design

This study was designed as a randomised controlled study. The patients were randomly divided by a computer code into two groups. Group A (treated group) included children who were given the tested product (a tablet containing SsM18) once a day for 3 months; group B (control group) included children who did not receive any treatment.

Before beginning the study, all the children had their teeth professionally cleaned with an abrasive paste to remove BSs. After polishing, a professional fluoride treatment was administered by the application of fluoride foam. They also received instructions on oral hygiene. The roll-on technique was chosen as it is easier to learn and less time-consuming compared to other complex teethbrushing techniques. Each child was given a new toothbrush and fluoride toothpaste at the beginning of the study. The children were forbidden to drink tea, fruit juice and other beverages containing tannin during the period of the study. The subjects also had the possibility to communicate daily with the physicians responsible for the study in order to report their medical conditions, probiotic tolerability or any possible side effects linked to the probiotics.

The assessment of BSs was done by two calibrated clinicians after 3 months (T1) and after 6 months (T2). The presence of BSs was recorded as ‘present’ or ‘absent’ independently from the number of teeth affected.

The children’s levels of oral hygiene were clinically assessed according to the criteria of simplified oral hygiene index (OHI-s) by Greene and Vermillion^12^ with a mouth mirror and explorer, during the first visit (T0), after 3 months (T1) and after 6 months (T2).

### Tested Products

*S. salivarius* M18 (IDA classification: DSM 14865), also named by the manufacturer as BLIS M18 (BLIS Technologies, Dunedin, New Zealand), was formulated as a slowly dissolving oral tablet by SIIT (Trezzano S/N, Italy) and notified as a nutritional supplement to the Italian Ministry of Health as Carioblis by Omeopiacenza (Pontenure, Italy), according to the provisions of law 169 of 2004, on 19 July 2013 (notification number 69163). The preparation of Carioblis used in our research contained no less than 1 billion colony-forming units (CFU)/tablet of *S. salivarius* strain M18.

### Treatment Protocol

Starting from day 0 to 90, one tablet of Carioblis was administered to each subject every night, just before going to sleep. The tablet was allowed to slowly dissolve in the oral cavity, without biting or swallowing. Saliva production is typically reduced in the evening hours and this improves the effectiveness of oral colonisation.

In order to evaluate the level of subject adherence to the established protocol, the subjects were asked to return any unused product boxes and tablets. Acceptable adherence was considered to be the administration of not less than 95% of the allocated tablets.

### Ethical Considerations

All patients were informed about the research and signed an informed consent form to take part. The study protocol was approved by the local Ethics Committee and performed according to the Declaration of Helsinki.

## Results

### Characteristics of the Patients

**Fig 1:**
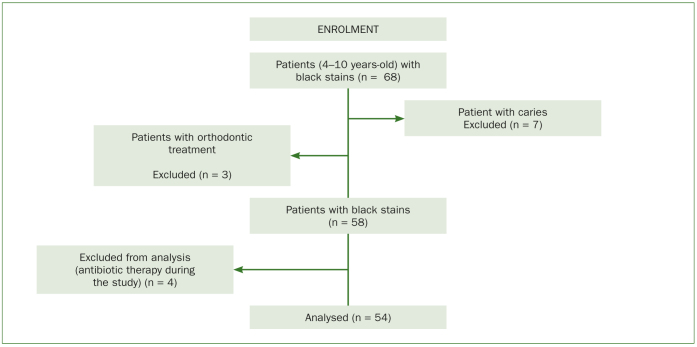
Enrolment flow-chart.

Among the 1227 children seen at the Pediatric Dentistry Department, a total of 68 patients (26 females and 32 males) showed BSs, of which 58 met the inclusion criteria (Fig 1). They were randomly assigned to two groups: group A (n = 29) and group B (n = 29). The mean age of the total sample was 7.0 + 1.35 years. There were no differences (p > 0.05) between the clinical and demographic characteristics of the two groups; therefore, the two groups were homogeneous (Table 1). During the study, 4 patients (1 belonging to group A and 3 to group B) were excluded because they started antibiotic therapy.

**Table 1:** Demographic and clinical characteristics of the patients

	Group A (n = 28)	Group B (n = 26)	Total (n = 54)
Male:Female	18:10	14:12	32:22
Mean age (range)	7.1 + 1.4 (4–10)	6.8 + 1.3 (4–10)	7.0 + 1.35 (4–10)

Mean + SD.

### Clinical Evaluation of OHI-s

No differences in the mean of OHI-s were observed between group A and group B, neither at T0, nor at T1. A statistically significant difference (p < 0.05) between the mean of the OHI-s in group A (1.06 + 1.16) and in group B (1.94 + 1.27) was found at T2 (Table 2).

**Table 2:** Presence of BSs (number of patients) and OHI-s values (mean + SD) at T0, T1 and T2 in the group A and in the group B

	GROUP A (n = 28)	GROUP B (n = 26)	
**Black stains** *(no. of patients)*			
T0	0	0	-
T1	6	13	p = 0.02*
T2	9	14	p = 0.1
**OHI values** *(mean + SD)*			
T0	1.95 + 0.97	1.67 + 1.15	p = 0.3
T1	1.77 + 1.23	1.81 + 1.17	p = 0.4
T2	1.06 +1.16	1.94 + 1.27	p = 0.02 *

*ANOVA test, p value < 0.05.

### Clinical Evaluation of BSs

After 3 months (T1), BSs were detected in 6 of the 28 children (21.2%) from group A and in 13 out of the 26 (50%) children from group B (p < 0.05). After 6 months (T2), BSs were detected in 9 out of the 28 (32.1%) children from group A and in 14 of the 26 (53.8%) children from group B (p > 0.05) (Table 2).

## Discussion

BSs are characteristic pigmented deposits that may occur at any age but seem to peak in childhood with a decrease in prevalence during pubescence and adulthood.^21^ Even if not correlated with caries, BSs represent an aesthetic problem, especially in the parents’ eyes. Nevertheless, BSs can be an annoyance for the dental hygienist, especially when they are deposited on roughened or pitted areas of the tooth. Currently, removal through professional oral cleaning is the only treatment but they tend to reform despite good personal oral care. Prevention of BSs could be a new challenge for the clinician.

This study evaluated the efficacy of *S. salivarius* M18 in the prevention of the reformation of BSs in children. A limit of this study was that the control group did not assume a placebo tablet. However, before the beginning of the study, all the children belonging both to the study group and to the control group underwent a professional oral hygiene process, received instruction on oral care and were forbidden to drink any beverages able to stain their teeth.

Our results show that the assumption of *S. salivarius* M18 for 3 months significantly reduced the reformation of BSs in children for that period without affecting its manifestation in the successive quarter. No differences in terms of level of personal oral hygiene were found between the two groups during the first 3 months. During the succeeding 3 months, the oral hygiene was significantly better maintained by the children belonging to group A, possibly due to the major compliance of the patients who participated to the active phase of the study.

The reason why a probiotic intake of *S. salivarius* M18 – which causes a new oral bacterial equilibrium – inhibits the formation of BSs should be further studied.

BSs are different than other forms of dental plaque due to their content of insoluble iron salts and high levels of calcium and phosphate.^13,18,25^ A possible reason for this condition might be a different, characteristic and relatively stable oral microflora. Various studies reported the association between BSs and *Actinomycetes*.^12,18,23^ The overgrowth of *Leptotrichia* and *Fusobacterium*, belonging to the bacterial phylum *Fusobacteria*, in plaque might be a cofactor to the formation of these pigmentations.^18,22,24^ *Fusobacterium* spp. can co-aggregate with black-pigmented anaerobes (*Porphyromonas gingivalis* and *Prevotella nigrescens*).^3,18^ Similarly, co-infecting or co-culturing *Pseudomonas aeruginosa* can also induce pigment production from *Staphylococcus aureus*.^1,18^However, how microbial assemblies in both saliva and plaque are linked to pigment production (represented by black extrinsic stain) and to low levels of caries remains largely unknown.

The *S. salivarius* strain M18 exhibits a particular bacteriocin and enzymatic profile, secreting A2, 9, MPS and *M. bacteriocins* together with urease and dextranase enzymes.^26^ Salivaricin A2, MPS and 9 are all plasmid-encoded and capable of inhibiting the growth of *Streptococcus pyogenes*. Salivaricin A2 and 9 are endowed with antipyogenes activity. Salivaricin MPS is active against *Corynebacterium* spp. and against *Streptococcus sanguinis*, which is relevant for the balance of the oral microbiota.^2^ M18 is directly active against *Actinomyces naeslundii* and *Actinomyces viscosus.*^5,9^

Salivaricin M is responsible for inhibiting mutans *Streptococci* and *Actinomyces*^26^; this could possibly be the mechanism which prevents the reformation of BSs.

Even if further studies on a wider sample of patients are still needed, based on our results, the M18 strain can be considered a smart nutraceutical tool to counteract BSs formation.
